# Heat Stress Induces Shifts in the Rumen Bacteria and Metabolome of Buffalo

**DOI:** 10.3390/ani12101300

**Published:** 2022-05-18

**Authors:** Zichen Wang, Kaifeng Niu, Hossam E. Rushdi, Mingyue Zhang, Tong Fu, Tengyun Gao, Liguo Yang, Shenhe Liu, Feng Lin

**Affiliations:** 1 College of Animal Science and Technology, Henan Agricultural University, Zhengzhou 450046, China; shirleywang2020@126.com (Z.W.); zmy1978339762@163.com (M.Z.); futong2004@126.com (T.F.); dairycow@163.com (T.G.); 2 College of Animal Science and Technology, Huazhong Agricultural University, Wuhan 430000, China; nkf_19930806@163.com (K.N.); yangliguo2006@foxmail.com (L.Y.); 3 Department of Animal Production, Faculty of Agriculture, Cairo University, Giza 12613, Egypt; hosamrushdi@agr.cu.edu.eg

**Keywords:** buffalo, heat stress, rumen fermentation, high-throughput sequencing, metabolomic

## Abstract

**Simple Summary:**

Heat stress is a major factor in causing substantial losses to the livestock industry, due to its negative effects on milk production, fertility, health and welfare. As the second largest diary producing species, buffalo face challenges from heat stress. To explore the nutritional metabolic mechanism of rumen bacteria in buffalo under hot enviroments, this study used 16S rDNA technology and metabolome analysis to identify heat stress-related bacteria, metabolites and their pathways. Our findings showed that heat stress increased respiratory rate and skin temperature, and decreased rumen volatile fatty acids. The structures of rumen bacterial communities at different levels significantly changed and a total of 32 metabolites mainly involved in gluconeogenesis were altered due to heat stress. Overall, this study improves our understanding of ruminal bacterial and metabolic alterations of buffalo exposed to heat stress.

**Abstract:**

Exposure to the stress (HS) negatively affects physiology, performance, reproduction and welfare of buffalo. However, the mechanisms by which HS negatively affects rumen bacteria and its associated metabolism in buffalo are not well known yet. This study aimed to gain insight into the adaption of bacteria and the complexity of the metabolome in the rumen of six buffalo during HS using 16S rDNA and gas chromatography metabolomics analyses. HS increased respiratory rate (*p* < 0.05) and skin temperature (*p* < 0.01), and it decreased the content of acetic acid (*p* < 0.05) and butyric acid (*p* < 0.05) in the rumen. Omics sequencing revealed that the relative abundances of *Lachnospirales*, *Lachnospiraceae*, *Lachnospiraceae_NK3A20_group* and *Clostridia_UCG-014* were significantly (*p* < 0.01) higher under HS than non-heat stress conditions. Several bacteria at different levels, such as *Lactobacillales*, *Streptococcus*, *Leuconostocaceae* and *Leissella*, were significantly (*p* < 0.05) more abundant in the rumen of the non-heat stress than HS condition. Thirty-two significantly different metabolites closely related to HS were identified (*p* < 0.05). Metabolic pathway analysis revealed four key pathways: D-Alanine metabolism; Lysine degradation, Tropane; piperidine and pyridine alkaloid biosynthesis; and Galactose metabolism. In summary, HS may negatively affected rumen fermentation efficiency and changed the composition of rumen community and metabolic function.

## 1. Introduction

Buffalo, as the second largest producers of meat and milk, are mainly distributed in tropical and subtropical regions of Asia [1]. Buffalo have a higher feed conversion efficiency and higher nutritive value of milk compared to dairy cows [2]. Buffalo present dark skin and sparse hair phenotypes, making the animals highly susceptible to absorb large amounts of solar radiation [3]. In addition, the sweat glands of buffalo are underdeveloped [4]. For this reason, buffalo have a poor heat dissipation capacity that makes them highly restricted to habitat requirements and considerably susceptible to global warming [5]. With the increasing impacts of the climate change, the issue of heat stress (HS) is likely to become worse for buffalo. The temperature-humidity index (THI) has been popularly used to indicate degree of HS. Heat stress in buffalo begin at THI 72, beyond which buffalo are unable to maintain heat balance, causing low dry matter intake (DMI) and high respiratory rate [6]. When the THI exceeds 75, animal productivity, fertility and health have been strongly affected, which subsequently cause economic losses in the dairy industry [7,8].

Most modern farms adopt measures including physical cooling and nutritional regulation to alleviate the impacts of HS on buffalo. However, these strategies may not eliminate completely the negative effects of HS due to animal-related factors including genetics, hair coat and metabolic heat production. With the development of modern molecular genetics technology, genomics techniques have attracted the vision of researchers. Metabolomics has been successfully applied for filtering out biomarkers for milk quality, energy metabolism, and rumen health in dairy cows [9,10]. Previous reports have applied macrobiotics techniques and found that the rumen microbial diversity of cows changes under the influence of HS [11]. Due to the high metabolic heat production with rumen fermentation, buffalo are more susceptible to the negative effects of HS compared with other farm animals [12]. As bioreactors, the rumen has highly dense and diverse microbial populations, which play an important role in buffalo health and metabolism [13]. The knowledge of rumen bacteria and metabolome could assist to better understand the effect of hot environment on animal productivity, physiology and immune activity. In addition, the identification of biomarkers may provide valuable tools to diagnose the incidence of HS in buffalo. Therefore, the objective of this study was to compare the changes in physiology, rumen microbes and their metabolites in buffalo under both HS and non-heat stress (NHS) conditions, so as to further understand how buffalo differentially respond to HS that ultimately could assist in formulating effective strategies to reduce the adverse effects of HS and provide a theoretical basis for improving milk production performance using means such as feeding management.

## 2. Materials and Method

### 2.1. Animal Management

Buffalo in this experiment were primarily selected based on days in milk (142 ± 11.1), and finally, six healthy second-parity buffalo of similar body weight (647 ± 24.48 kg) and age (49 ± 1.48 months) were selected. The experiment was conducted at Hubei Prime Cattle Husbandry Co., Ltd. (Jingmen, China) during June and August of 2021 as NHS and HS condition for seven days, respectively. The same management system was applied along the whole experimental period. All the animals were housed in individual pens with free access to water. Total mixed rations (TMR) were fed twice daily and were offered to ensure 10% residual feed. Feed intake was calculated for each cow during the trial. Samples of feed stuffs and TMR were collected on days 1, 4, 7 for each cow for NHS and HS period. Then, feed ingredients and composition were determined according to Association of Official Analytical Chemists (AOAC) standards in the laboratory [14]. Detailed composition and nutrient content of the diets are presented in Table 1.

### 2.2. Sample Collection and Measurement

Two distinct time points, as HS and NHS conditions, were determined based on THI. The temperature and humidity were continuously recorded every hour during the trial for calculating THI, which was calculated by using the following formula: THI = (1.8 × Tdb + 32) − [(0.55 − 0.0055 × RH) × (1.8 × Tdb − 26.8)], where Tdb is the dry-bulb temperature (°C), and RH is the relative humidity (%) [4]. Simultaneously, physiological parameters including respiratory rate, rectal temperature and skin temperature were measured three times daily (8:00 a.m., 12:00 p.m. and 17:00 p.m.) throughout the experimental period according to the method described by [8]. Buffalo were milked twice per day, and milk yield was recorded at each milking for each cow. Milk samples were collected during evening milking at day 7 and analyzed for the individual cow for fat, protein, lactose, milk urea nitrogen and SCC at the laboratory of Hubei Dairy Herd Improvement Center. Blood samples from the jugular vein of each cow were taken at 12:00 p.m. on the last day of the experimental period. A 10 mL blood collection tube was used for plasma separation. Then, blood samples were centrifuged at 3500× *g* for 10 min and stored at −20 °C until performing the analyses of heat shock protein 70 (HSP70) and heat shock protein 90 (HSP90). The serum concentrations of HSP70 and HSP90 were determined by enzyme immunoassay-ELISA commercial reagent kits (Shanghai Enzyme-linked Biotechnology Co., Ltd., Shanghai, China). The respective intra- and inter-assay coefficients of variations were less than 10%. 

As previously described by [15], rumen fluid samples were extracted from the rubber end of the rumen tube connected with a syringe posterior on the last day of the NHS and HS periods, prior to morning feeding. The oral stomach tube was inserted to a depth of 200 cm to ensure that it reached the central rumen, thus obtaining representative rumen fluid samples [16]. The first two extractions were discarded for minimizing saliva contamination. The pH of rumen fluid was measured immediately after sample collection, and then stored in sterile tubes and snap-frozen in liquid nitrogen before storage at −80 °C for subsequent DNA extraction and analysis. Total volatile fatty acids (TVFA) were determined by ion chromatography using Dionex ICS-3000 ion chromatograph following the procedure proposed by [17,18]. The concentration of NH_3_N was measured using visible-light spectrophotometry (Scientific BioMate 3s, Thermo, Waltham, MA, USA). 

### 2.3. DNA Extraction, 16S rDNA Sequencing and Sequences Analysis

After thawing, microbial genomic DNA was extracted from 1 mL rumen samples using a commercial DNA extraction kit (OMEGA Bio-Tek, Norcross, GA, USA), in accordance with the manufacturer’s instructions [19]. DNA concentration was determined using a spectrophotometer (Nanodrop, Thermo Fisher Scientific Oxoid Ltd., Basingstoke, UK). The amplification of 16S rDNA V3 to V4 regions was conducted using the forward primer 338F (5′-ACTCCTACGGGAGGCAGC-3′) and the reverse primer 806R (5′-GGACTACHVGGGTWTCTAAT-3′) [20]. The 5′ end of the primers in each sample was marked with specific barcodes. The specific PCR products were detected and quantified using the QuantiFluor™-ST blue fluorescence quantitation system (Promega Co., Ltd., San Diego, CA, USA), followed by mixing in equal amounts. The detailed PCR procedure applied was according to that described by [20]. The amplicon library was quantified using an Agilent 2100 Bioanalyzer (Agilent Technologies, Santa Clara, CA, USA) and assessed with the Hieff NGSTM Library Quantification Kit for Illumina (Kapa Biosciences, Woburn, MA, USA). After the constructed library was qualified, the Illumina PE250 system was used for sequencing (Shanghai Majorbio Bio-pharm Technology Co., Ltd., Shanghai, China). Raw sequencing reads have been deposited in the NCBI Sequence Read Archive (SRA) under Bio Project PRJNA793724.

Following sequencing, raw sequences were filtered and classified using MOTHUR (v1.30.2). The sequence shorter than 200 bp, or with a homopolymer longer than 8 bp and the raw reads containing ambiguous bases were removed to ensure high accuracy for the subsequent analysis. Non-repetitive sequences (excluding single sequences) were clustered for operational taxonomic unit (OTU) following 97% similarity (UPARSE, version 7.0.1090), based on a representative sequence of the resulting OTU. Analyses of bacterial abundances and diversity index, and the representative sequence of each OTU were aligned against the Silva database (Release138 http://www.arb-silva.de, accessed on 15 October 2021). The overall microbiotas shaped by the two different temperature conditions throughout the experimental period were measured using principal component analysis (PCA) and principal co-ordinates analysis (PCoA) by means of QIIME software (v1.9.1)

### 2.4. Metabolomics Profiling for Rumen Fluid and Data Analysis

Rumen samples were prepared to be pretreated, extracted and derivatized according to the method described by [21]. Quality control samples were prepared to evaluate the stability of the analytical system. Furthermore, the quality control samples with relative standard deviation ≥ 30% were deleted. In brief, a 100 μL biofluid sample was mixed with 300 μL methanol, followed by adding 50 μL L-2-chlorophenylalanine, and then the mixture was vortexed. Subsequently, all sample mixtures were kept at −20 °C for 20 min and centrifuged for 15 min (14,000× *g*, 4 °C). The extracts were dried using a vacuum concentrator, then a volume of 80 μL of o-methyl hydroxylamine hydrochloride was added, and finally the solution was vortexed again and maintained for further analysis.

The gas chromatography metabolomics (GC/MS) data analysis was conducted by integrating each resolved chromatogram peak. The metabolite annotation and data pretreatment were conducted on the free online platform of Majorbio Cloud Platform (www.majorbio.com, accessed on 15 October 2021). After raw data collection, orthogonal projections to latent structures discriminant analysis (OPLS-DA) were carried out by ropls (R packages, Version 1.6.2). The metabolites were identified and confirmed by using Kyoto Encyclopedia of Genes and Genomes (KEGG), whereas MetaboAnalyst 4.0 was performed to obtain the relevant pathways [22]. Furthermore, the significantly differential metabolites were screened using variable importance in projection (VIP) scores (VIP >1) from the OPLS-DA, *p*-values (*p* < 0.05) and the fold change (FC) (log_2_ FC > 1 or <−1).

### 2.5. Statistical Analysis

The correlations between altered rumen bacteria and various heat stress response indices, including the physiological parameters, animal performance and rumen fermentation were assessed by Pearson correlation analyses, where *p*-value of less than 0.05 was considered statistically significant. Physiological parameters, blood parameters, animal performance and rumen fermentation were tested for normality using Shapiro–Wilk’s test, and the data conformed to a normal distribution. Since SCC is a skewed distribution, convert it to somatic cell score (SCS) according to the formula for the analysis: SCS = Log_2_ (SCC/100) + 3 [23]. Then, the data were statistically analyzed by the linear mixed model. The statistical model included the fixed effects of factors (the HS and NHS conditions) and covariates (DIM and DMI); the random effect of the animal. Pearson correlation analyses, normal distribution test and linear mixed model were performed using SPSS 25.0 software (IBM Inc., Chicago, IL, USA). Significant differences were declared at *p* < 0.05, whereas *p* < 0.01 was considered highly significant. The results of these analyses were expressed as means and standard error of the means (SEM).

## 3. Results

### 3.1. Physiological Parameters, Animal Performance and Rumen Fermentation 

Measures of THI recorded in June as NHS condition (THI = 70.31), and in August, as HS condition (THI = 80.77) indicated that the buffalo individuals were exposed to HS during August. As shown in Table 2, respiratory rate (*p* < 0.01) and skin temperature (*p* < 0.05) were increased in the HS condition compared with the NHS condition. Regarding rumen fermentation, values of acetic acid (*p* < 0.05), butyric acid (*p* < 0.05), TVFA (*p* < 0.05) and pH (*p* < 0.05) were lower in the HS condition as compared with the NHS condition.

### 3.2. Rumen Bacteria Diversity and Composition

**Figure 1:**
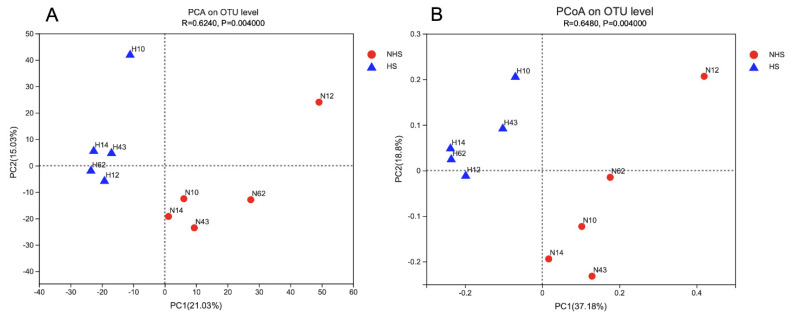
Beta diversity of ruminal microbiota using principal component analysis (**A**) and principal co-ordinates analysis (**B**). NHS, non-heat stress condition; HS, heat stress condition.

High throughput sequencing of the 16S rDNA gene revealed that a total of 677,955 raw reads were obtained, whereas 39,870 OTU were obtained by performing OTU clustering on nonrepetitive sequences according to 97% similarity. The detailed sequence information is provided in Table S1. As shown in Table 3, Good’s coverage values for the two periods were greater than 0.99, indicating good sequencing depth for analysis of the rumen microbiota. Microbial community richness and diversity, as represented by Sobs, ACE and Shannon diversity indices of the microbial communities in the rumen of buffalo, showed no significant difference between NHS and HS conditions. Additionally, PCA and PCoA based on Bray–Curtis distance were performed to compare the composition of bacterial communities under both NHS and HS conditions revealing that the bacterial communities were obviously separated by HS (Figure 1). 

**Figure 2:**
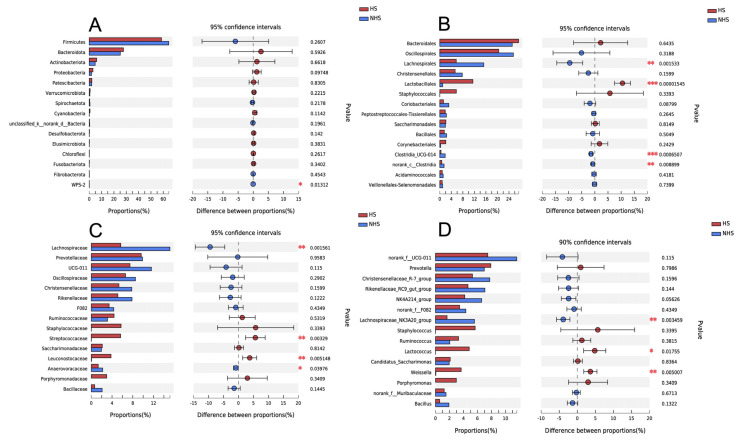
Rumen bacteria affected by heat stress at different taxonomical levels. (**A**) phylum (**B**) order level (**C**) family level (**D**) genus level. * represents *p* < 0.05, ** represents *p* < 0.01, *** represents *p* < 0.001. NHS, non-heat stress condition; HS, heat stress condition.

Ruminal bacterial community composition and structure were analyzed at different taxonomical levels. At the phylum level (Figure 2A), most sequences were assigned to *Firmicutes* (58.56–64.41) and *Bacteroidota* (25.14–27.73). Four predominant taxa at order level including *Lachnospirales*, *Lactobacillales*, *Clostridia_UCG-014*, and *norank_c__Clostridia* were significantly shifted (*p* < 0.01) during the period of HS (Figure 2B). Significant shifts were also detected at family level (Figure 2C). The relative abundances of *Lachnospiraceae* and *Anaerovoracaceae* were significantly (*p* < 0.05) higher in HS condition, whereas *Streptococcaceae* and *Leuconostocaceae* were significantly (*p* < 0.01) lower in HS condition. In addition, HS resulted in a decrease (*p* < 0.05) in genus level in the relative abundances of *Lactococcus* and *Weissella*, and a significant (*p* < 0.01) elevation in the relative abundances of *Lachnospiraceae_NK3A20_group* (Figure 2D). 

### 3.3. Identification of Different Metabolites and Metabolic Pathways

**Figure 3:**
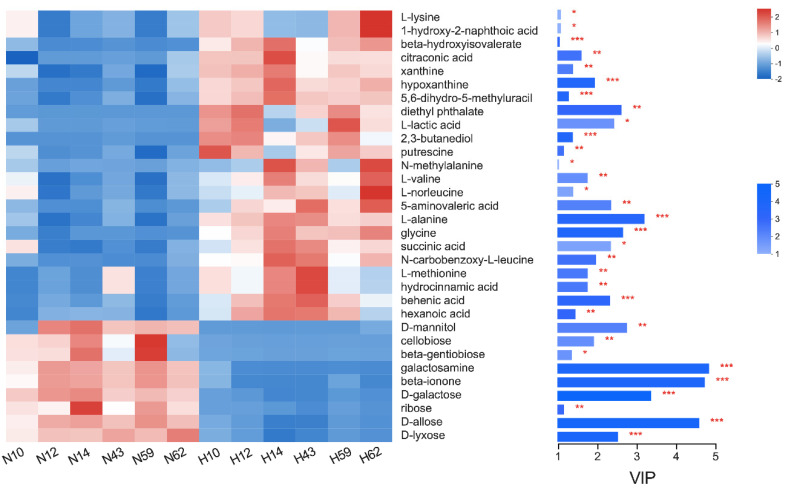
Differential metabolites in rumen fluid of buffalo under non-heat stress and heat stress conditions. The heatmap is the metabolite expression and the right side is the metabolite variable importance in projection (VIP) bar graph. The color of the bar indicates the significance of the difference between the two groups of metabolites, * represents *p* < 0.05, ** represents *p* < 0.01, *** represents *p* < 0.001. N, non-heat stress condition; H, heat stress condition.

**Figure 4:**
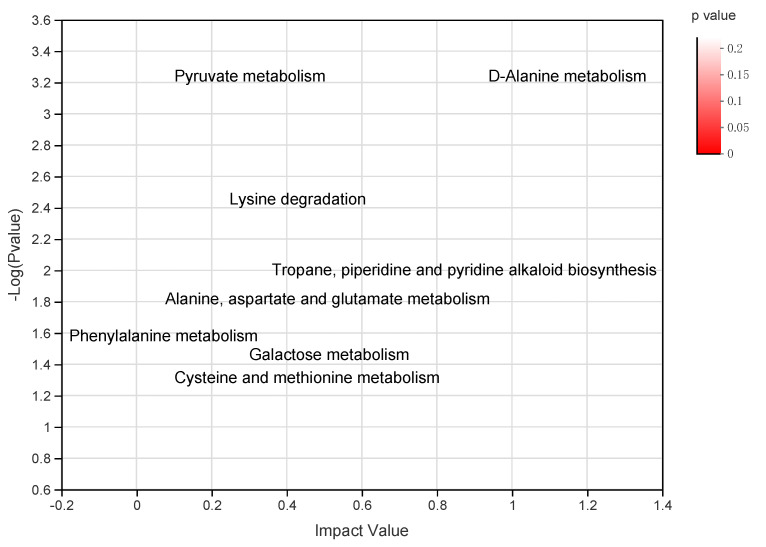
Key differential metabolic pathways characterize in the rumen fluid of buffalo under non-heat stress and heat stress conditions. The *x*-axis represents the pathway impact, and *y*-axis represents the pathway enrichment.

The OPLS-DA analysis of GC-MS metabolic profiles of rumen fluid showed a separation between the NHS and HS (Figure S1). For the rumen metabolome, we analyzed the 197 compounds identified in the present study. After *t*-test and VIP filtering for the relative concentrations of rumen metabolites, the comparison analysis revealed that the concentrations of 9 metabolites were significantly (*p* < 0.05, VIP > 1) higher in the rumen of animals under HS compared to NHS conditions, whereas 23 metabolites were significantly (*p* < 0.05, VIP > 1) lower in the rumen of buffalo assigned to HS compared to NHS condition (Figure 3). The metabolome view map based on 32 differential metabolites revealed the enrichment of 8 pathways (*p* < 0.05). However, only 4 of them were characterized as significantly relevant pathways which had a pathway impact value higher than 0.1, involved in D-Alanine metabolism, Lysine degradation, Tropane, piperidine and pyridine alkaloid biosynthesis, and Galactose metabolism (Figure 4).

### 3.4. Correlation between Rumen Bacteria, Metabolites and Fermentation Parameters

**Figure 5:**
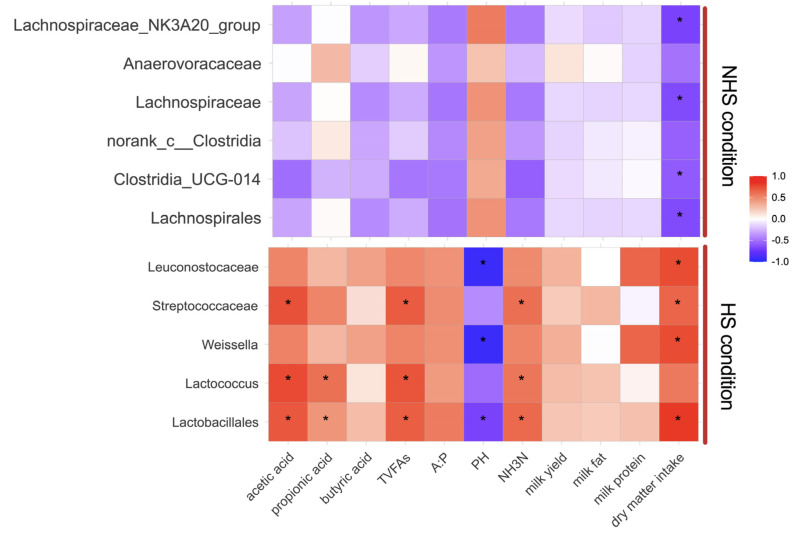
Effect of heat stress on correlation analysis between rumen bacteria and heat stress response indices including physiological and blood parameters, animal performance and rumen fermentation were assessed by Pearson correlation analyses. NHS, non-heat stress condition; HS, heat stress condition. Red indicates a positive correlation; the blue indicates a negative correlation; asterisks denote significant differences at *p* < 0.05.

To explore the relationships between rumen fermentation, physiological and blood parameters, animal performance and rumen bacteria, Pearson correlation coefficients were calculated for the HS and NHS conditions (Figure 5). Significantly negative correlations were found between *Lachnospirales*, *Lachnospiraceae_NK3A20_group*, *Lachnospiraceae and Clostridia_UCG-014* with dry matter intake (r < 0.5, *p* < 0.05). We also detected that *Lactobacillales*, *Streptococcaceae* and *Weissella* were positively correlated with NH_3_N, acetic acid and VFA (r < 0.5, *p* < 0.05).

## 4. Discussion

Heat stress is a severe challenge for buffalo. Previous studies have found that farm animals display differential HS responses at the ambient temperature [24,25,26]. In the present study, the significant differences between NHS and HS conditions in terms of their effects on respiratory rate and skin temperature reveal buffalo sensitivity to hot environments. Due to fewer sweat glands, buffalo lose 88% of the internally produced heat, whereas the remaining body heat dissipates from the skin surface by sweating [6]. Previous studies in dairy cows have found a reduction in milk yield by 30–40% due to HS, and this decrease in milk production can be partly attributed to reduced feed intake [27,28]. In addition, an increase in rectal temperature by 1 °C under HS conditions was sufficient to reduce the performance of dairy cows [29]. However, the negative effect of heat stress on milk yield and feed intake in dairy cows is not reflected on buffalo individuals in our study. We speculate that this discrepancy may be attributable to the fact that higher producing dairy cows with greater growth and metabolic activity compared to buffalo, the body heat load is increased, thus amplifying heat strain in dairy cows [30]. The difference in reaction to HS may be also related to variation in heat tolerance ability among between different breeds and individuals. Further investigations trials are needed to expand the sample size and compare the response to heat stress among individuals with different heat tolerance in the same breed. HS altered buffalo rumen fermentation in the present study, including a decrease in acetic and butyric acid, which are precursors of a diverse range of compounds in the body especially fatty acids and total cholesterol [31]. Previous studies have documented that differential basic metabolism of VFA in rumen caused variation in milk quality, as acetic acid affects milk fat synthesis, and propionic acid is involved in the biosynthesis of milk protein and lactose [32,33]. 

Rumen fermentation has a dynamic response to alterations in the rumen microbiome [34]. The 16S rDNA gene sequencing analysis (V3–V4) used in this study only focused on bacteria, which are the most predominate and diverse among the ruminal microbiota (archaea, fungi, protozoa and bacteria) [35]. The rumen is a major habitat for numerous species of microbes, where fluctuations in the number and composition of rumen bacteria reflect changes in nutrients and rumen physiological environments [36]. In the current study, HS had no significant influence on the alpha diversity of the rumen bacteria. On the contrary, the analysis of β-diversity showed noticeable changes in the bacterial community composition owing to HS. We found that the bacteria of the rumen were mainly composed of *Firmicutes* and *Bacteroidota* (the relative abundance > 5%), which is in agreement with previous studies in buffalo [37]. Regarding the relative abundances of major order and family, we found that the relative abundances of *Lactobacillales*, *Streptococcus* and *Leuconostocaceae* were higher under the HS compared with NHS condition, and lactic acid is the main end-product of their characteristic heterofermentative carbohydrate metabolism [38]. Of note, the absorption rate of lactic acid from the rumen epithelium is slow, thereby reducing rumen pH [31]. Corresponding to this, the present study also showed that HS significantly decreased pH of rumen, which was negatively correlated with the relative abundances of *Lactobacillales* and *Leuconostocaceae.* In addition, *Lactobacillales* was positively correlated with acetic acid and VFA in our study. Taken together, *Lactobacillales* and *Leuconostocaceae* may play a major role in the decline of rumen pH due to HS, and further affect the living environment of other bacterial types. *Leuconostocaceae* species are used for the production of fermented milk, butter, and cheese together with *Lactococcus*, *Streptococcus* and *Lactobacillus* species [39]. However, what roles the bacterium plays in determining milk quantity and quality under HS and their underlying mechanisms are still unclear. 

Acetic and propionic acids are responsible for the formation of fatty acids and cholesterol, which are related to milk fat content, and failure to ensure glucose production when propionic acid is insufficient leads to insufficient energy supply [40,41]. As reported earlier, acetic-acid-producing bacteria and propionic-acid-producing bacteria such as *Christensenellaceae_R-7_group*, *Rikenellaceae_RC9_gut_group* and *Ruminococcus* may contribute to HS responses in cows by increasing heat production in the rumen metabolism [34,42]. However, these genera of bacteria did not differ significantly between the two periods investigated in the present study. It could be concluded that increasing the numbers of lactate-producing bacteria resulted in extra production of lactate and a reduction in the rumen pH under HS condition, which is consistent with the findings in dairy cows [31]. Additionally, *Weissella*, a genus of Gram-positive bacteria under *Leuconostocaceae* family, can synthesize various bioactive compounds resulting in a better bioavailability, as well as producing glucan [43]. The microbial composition of the rumen is influenced by various factors such as breed, feeding regimen, age, genetics and sex [44]. The complex relationships between the dynamic changes in rumen microbiota induced by HS, on one side, and feed utilization and production efficiency, on the other side, in domestic buffalo require further investigation. Since the effect of HS on bacteria, archaea, fungi, and protozoa of rumen microbiota may vary differently [45], the microbial diversity and the host-microbiota interaction due to HS also requires further research.

Metabolomics play important roles in understanding physiological and biochemical status of animals [46]. In this study, GC-MS was used to detect the changes in ruminal metabolites from buffalo under NHS and HS conditions. Alanine is the main precursor of glucose production via gluconeogenesis [47]. It has been reported that accumulation of alanine as a major amino acid is increased under HS conditions [48,49]. Our finding is strongly consistent with L-alanine significant increase, indicating the synthesis of glucose by buffalo during HS period and that the cellular energy matrix is insufficient. In ruminants, the gluconeogenic pathway is active and the rumen microorganisms break down cellulose to generate VFA, which are converted to succinyl CoA to participate in the gluconeogenic pathway for glucose synthesis [50,51]. In addition, lactic, citric and succinic acids with greater content in the HS condition can be converted to oxaloacetic acid and then enter the gluconeogenic pathway [52]. Although the high content of glucogenic amino acids (lysine, valine, alanine, glycine and L-methionine) are precursors of milk protein in the HS condition, they are preferentially used to generate tricarboxylic acid intermediates (citric acid, succinic acid, etc.) to participate in the gluconeogenic pathway and amino acid metabolism under HS conditions to maintain energy balance [53,54,55]. We hypothesize that these amino acids are capable of alleviating the adverse effects of HS on buffalo, enhance immunity and respond to anti-oxidation. 

This study also deals with most influential pathways involved in significantly different metabolites. In the pyruvate metabolism pathway, on the one hand, pyruvate acts as the end product of glycolysis, and is dehydrogenated to lactate; on the other hand, pyruvate is converted into non-essential amino acids by the action of transaminases and participates in the tricarboxylic acid (TCA) cycle through generating acetyl CoA [56], and then produces adenosine triphosphate, thus providing energy required for milk protein synthesis in dairy cattle [9]. The high concentration of lactic acid and non-essential amino acids in the HS condition of the current study supports the concept of energy supply to the buffalo body through the pyruvate metabolic pathway during the HS condition, suggesting that this pathway may contribute to more active redox reactions to produce more metabolic energy in response to HS. Furthermore, HS affects the metabolism of glucose through diverse pathways such as glycolysis, TCA cycle and galactose metabolism. HS also influences the metabolism of glutamic acid that is an important metabolite in the TCA cycle, leading to a significant decrease in intracellular glutathione levels as well as an increase in reactive oxygen species and oxidative stress [52]. The above-mentioned pathways broadly illustrate that amino acids and energy levels can maintain energy balance under the HS conditions and thus play a fundamental role in buffalo against HS. 

## 5. Conclusions

In general, HS impacted the physiological status of buffalo regarding an increase in respiratory rate and skin temperature. In addition, HS may negatively affected rumen fermentation efficiency and altered the bacterial community and metabolomic profiles in the rumen. Data analysis demonstrated that the structures of rumen bacterial communities at different levels significantly changed under the HS condition compared to NHS condition. The results showed that a total of 32 metabolites mainly involved in gluconeogenesis were altered due to HS. These findings provide new insights to better understand the impacts of HS on rumen fermentation and metabolic disorders. Nevertheless, further research is needed to validate the results of the present study. 

## Figures and Tables

**Table 1:** Feed ingredients and composition of the diets fed to experimental buffalo (*n* = 6).

Item	Content
Ingredient (%, DM basis)	
Silage corn	30
Rice straw	45
Peanut vine	10
Corn	7
Rice bran	2.5
Soybean meal	3
Wheat bran	2.5
Mineral lick ^1^	
Nutrient composition	
DM, %	67.50
NDF, % of DM	32.74
ADF, % of DM	20.05
CP, % of DM	11.60
Ca, % of DM	0.70
P, % of DM	0.50
GE, Mcal/kg of DM	7.58

Note: provided by Hubei Prime Cattle Husbandry Co., Ltd., Jingmen, China. ^1^. The ingredients of licking bricks are urea, Nacl, molasses, minerals and multivitamins (Cangzhou Haili brick Co., Ltd., Cangzhou, China). Abbreviations: DM = dry matter; NDF = neutral detergent fiber; ADF = acid detergent fiber; CP = crude protein; GE = gross energy.

**Table 2:** Differential response in physiological parameters, blood parameters, animal performance, and rumen fermentation for buffalo under non-heat stress (NHS; *n* = 6) and heat stress (HS; *n* = 6) conditions.

Index	NHS	HS	SEM	*p*-Value
Physiological parameters				
Respiratory rate (bpm)	46.39	66.27	6.74	0.005
Rectal temperature (°C)	38.74	38.80	0.14	0.831
Skin temperature (°C)	35.66	38.22	0.71	0.010
Blood parameters				
HSP70 (ng/mL)	395.91	568.79	117.77	0.126
HSP90 (ng/mL)	236.84	376.33	113.90	0.525
Animal performance				
DMI (kg/d)	13.03	11.57	1.29	0.456
Milk yield (kg/d)	4.10	2.93	1.72	0.480
Fat (%)	8.66	7.71	1.07	0.415
Protein (%)	4.76	4.47	0.49	0.064
Milk urea nitrogen (mg/dL)	14.64	20.48	3.42	0.060
SCS	3.68	3.88	1.26	0.581
Rumen fermentation				
pH	6.85	6.63	0.15	0.031
Acetic acid (mmol/L)	44.72	30.07	6.38	0.030
Propionic acid (mmol/L)	13.29	11.22	1.86	0.467
Butyric acid (mmol/L)	9.38	7.98	0.81	0.016
TVFA (mmol/L)	67.39	49.27	8.35	0.038
A:P	3.36	2.76	0.32	0.089
NH_3_N (mg/dL)	5.33	10.76	2.49	0.059

Abbreviations: bpm = breath per minute; HSP = heat stress protein; DMI = dry matter intake; SCS = somatic cell score; TVFA = total volatile fatty acid; A:P = acetic acid to propionic acid ratio. Note: Physiological parameters were measured three times daily (08:00 a.m., 12:00 p.m. and 17:00 p.m.); DMI and milk yield were recorded twice daily; milk samples were collected during evening milking at day 7 and analyzed for milk composition; blood and rumen fluid samples were collected on the last day of the experimental period, prior to morning feeding.

**Table 3:** Effects of HS on richness and diversity of rumen bacteria in buffalo (*n* = 6).

Index	NHS	HS	SEM	*p*-Value
Sobs	1487.40	1581.80	89.51	0.351
ACE	1738.79	1842.35	139.09	0.498
Shannon	5.43	5.68	0.15	0.170
Coverage	0.99	0.99	0.00	0.835

Abbreviations: NHS = non−heat stress; HS = heat stress; ACE = abundance−based coverage estimator.

## Data Availability

All the analyzed datasets in the current study are available from the corresponding author on reasonable request. The sequence data reported in this paper have been deposited in the NCBI database (BioProjec: PRJNA793724).

## Supplementary Materials

The following supporting information can be downloaded at: https://www.mdpi.com/article/10.3390/ani12101300/s1, Figure S1: Orthogonal partial least squares discriminant analysis (OPLS-DA) plot (A) and response permutation testing (B) of rumen metabolites in comparisons of the non-heat stress (NHS) and heat stress (HS) conditions; Table S1: Detailed sequence information of rumen samples of buffaloes using high-throughput sequencing
