# Supplementary material for: Heat Stress Induces Shifts in the Rumen Bacteria and Metabolome of Buffalo
Source: Animals (Basel). 2022 May 18;12(10):1300. doi: 10.3390/ani12101300 (PMC9137813; doi:10.3390/ani12101300)
Supplement: Supplementary file 1 [file animals-12-01300-s001.zip › animals-1636232-supplementary.pdf]

animals

Article

Heat Stress Induces Shifts in the Rumen Bacteria and Metabolome of Buffalo

Zichen Wang [1,†], Kaifeng Niu [2,†], Hossam E. Rushdi [3], Mingyue Zhang [1], Tong Fu [1], Tengyun Gao [1], Liguo Yang [2], Shenhe Liu [1,*] and Feng Lin [1,*]

[1] College of Animal Science and Technology, Henan Agricultural University, Zhengzhou 450046, China; shirleywang2020@126.com (Z.W.); zmy1978339762@163.com (M.Z.); futong2004@126.com (T.F.); dairycow@163.com (T.G.)
[2] College of Animal Science and Technology, Huazhong Agricultural University, Wuhan 430000, China; nkf_19930806@163.com (K.N.); yangliguo2006@foxmail.com (L.Y.)
[3] Department of Animal Production, Faculty of Agriculture, Cairo University, Giza 12613, Egypt; hosamrushdi@agr.cu.edu.eg
* Correspondence: liushenhe2015@163.com (S.L.); linfeng7207@163.com (F.L.)
† These authors contributed equally to this work.

Table S1. Detailed sequence information of rumen samples of buffaloes using high-throughput sequencing.

Sample	Seq_number	Base_number	Mean_length	Min_length	Max_length
H10	43571	18040789	414.054968	201	431
H12	59390	24765862	417.003906	253	434
H14	48017	19989670	416.304017	353	445
H43	60209	25025951	415.651331	277	437
H59	55790	23582750	422.705682	215	468
H62	51681	21509724	416.201776	216	505
N10	65293	27273589	417.710765	218	476
N12	60253	24863760	412.655967	365	452
N14	59704	24580669	411.708914	255	445
N43	70671	29492820	417.325636	209	481
N59	55066	22703242	412.291468	238	446
N62	48310	20005083	414.098178	248	448
Total	677955	281833909	415.642717	201	505

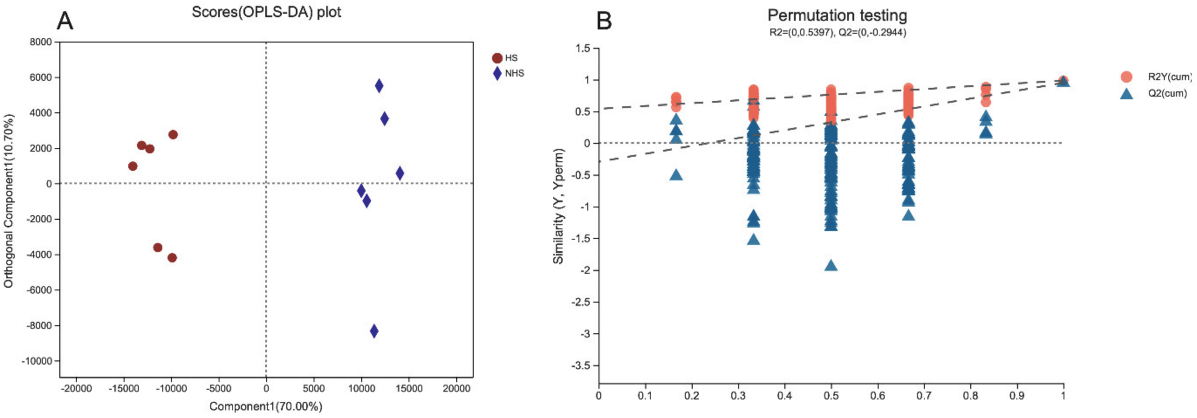

Figure S1. Orthogonal partial least squares discriminant analysis (OPLS-DA) plot (**A**) and response permutation testing (**B**) of rumen metabolites in comparisons of the non-heat stress (NHS）and heat stress (HS) conditions. R2Y (cum) and Q2 indicates the cumulative interpretation power and predictive power of the model, respectively.
